# Feasibility analysis of low-dose CT with asynchronous quantitative computed tomography to assess vBMD

**DOI:** 10.1186/s12880-023-01115-1

**Published:** 2023-10-06

**Authors:** Tingting Hu, Xingyuan Yang, Lei Gao, Ying Liu, Wei Zhang, Yan Wang, Xiaona Zhu, Xiangdong Liu, Hongran Liu, Xiaohui Ma

**Affiliations:** 1 Department of Radiology, Hebei Medical University Third Hospital, No. 139 Ziqiang Street, Qiaoxi District, Shijiazhaung, Hebei 050051 China; 2 Department of CT/MRI, Hebei Medical University Third Hospital, No. 139 Ziqiang Street, Qiaoxi District, Shijiazhaung, Hebei 050051 China; 3 Department of Endocrinology, Hebei Medical University Third Hospital, No. 139 Ziqiang Street, Qiaoxi District, Shijiazhaung, Hebei 050051 China; 4 Department of Vascular Surgery, Hebei Medical University Third Hospital, No. 139 Ziqiang Street, Qiaoxi District, Shijiazhaung, Hebei 050051 China

**Keywords:** Asynchronous quantitative computed tomography, Volumetric bone mineral density, Low-dose CT

## Abstract

**Background:**

To explore the feasibility of low-dose computed tomography (LDCT) with asynchronous quantitative computed tomography (asynchronous QCT) for assessing the volumetric bone mineral density (vBMD).

**Methods:**

416 women patients, categorized into 4 groups, were included and underwent chest CT examinations combined with asynchronous QCT, and CT scanning dose protocols (LDCT or CDCT) were self-determined by the participants. Radiation dose estimations were retrieved from patient protocols, including volume CT dose index (CTDIvol) and dose-length-product (DLP), and then calculated effective dose (ED). Delimiting ED by 1.0 mSv, chest CT examinations were categorized into 2 groups, LDCT group and CDCT group. vBMD of T12-L2 was obtained by transferring the LDCT and CDCT images to the QCT workstation, without extra radiation.

**Results:**

There was no difference of vBMD among 4 age groups in LDCT group (*P* = 0.965), and no difference in CDCT group (*P* = 0.988). In LDCT group and CDCT group, vBMD was not correlated to mAs, CTDIvol and DLP (*P* > 0.05), respectively. Between LDCT group and CDCT group, there was no difference of vBMD (*P* ≥ 0.480), while differences of mAs, CTDIvol and DLP.

**Conclusion:**

There was no difference of vBMD between LDCT group and CDCT group and vBMD was not correlated to mAs. While screening for diseases such as lung cancer and mediastinal lesions, LDCT combined with asynchronous QCT can be also used to assess vBMD simultaneously with no extra imaging equipment, patient visit time, radiation dose and no additional economic cost.

**Supplementary Information:**

The online version contains supplementary material available at 10.1186/s12880-023-01115-1.

## Background

A calibration phantom should be placed on the patient’s dorsal side during CT examination to assess trabecular volumetric bone mineral density (vBMD) in traditional quantitative computed tomography (QCT) [1, 2]. Asynchronous calibration QCT (asynchronous QCT) is a new tool to quantitatively assess trabecular vBMD without the use of calibration phantom during QCT examination. Trabecular vBMD assessed by QCT could be used to diagnose osteopenia and osteoporosis, osteopenia which defined as vBMD from 80 milligrams per cubic centimeter (mg/cm^3^) to 120 mg/cm^3^, and osteoporosis which defined as vBMD less than 80 mg/cm^3^, respectively [3–6]. Osteopenia or osteoporosis, which were characterized by low bone mass, microarchitectural deterioration and fragility fractures, is the growing clinical problem in older people, especially postmenopausal women [7, 8]. Previous studies have used conventional-dose CT (CDCT) combined with asynchronous QCT to measure vBMD, but few studies used low-dose CT (LDCT) [1, 5]. Low-dose CT (LDCT) of chest is feasible and common for early screening of lung cancer [9], also performed for other indications such as mediastinal lesions.

Previous study, taken European spine phantom (ESP) as the research object, showed that the vBMD measured by QCT with low-mAs was repeatable and accurate [10]. While due to the radiation dose, it is impossible to assess whether there was a difference in vBMD between CDCT and LDCT on the same patient at the same time. Ionizing radiation exposure is associated with a gradually increased risk of cancer. In order to avoid unnecessary radiation dose, scanning techniques of CT should be optimized and always comply with “as low as reasonably achievable” (ALARA) principle proposed by the International Commission on Radiological Protection [11, 12].

The purpose of our study was to explore the feasibility of LDCT with asynchronous QCT for assessing the vBMD, compared with vBMD measured by CDCT.

## Methods

### Patient population

We retrospectively reviewed a single-center database of QCT examination from January 2020 to April 2022 with approval from the local institutional review board (W2022-031-1). All participants provided written informed consent before CT examination. Women from 26 to 45 years old were included. Strictly speaking, in order to evaluate the feasibility of LDCT with asynchronous QCT for assessing the vBMD compared with CDCT, the participants should perform LDCT and CDCT at the same time. However, based on the principle of ALARA, this is not good for participants and is not ethical. Therefore, we compared the vBMD measured by LDCT and CDCT with asynchronous QCT in the two samples at the same age. In this case, the two samples must reflect the vBMD of the population at this age as far as possible. So, the factors affecting the vBMD should be excluded. Exclusion criteria included diabetes mellitus, smoking and drinking, history of paralysis, and history of malignant tumor, history of ovarian and/or uterine surgery, vertebral fracture and/or surgery, and suffering disease that influence bone metabolism including renal failure, hyperthyroidism and hyperparathyroidism and/or taking drugs affecting bone metabolism such as sex steroids, warfarin and bisphosphonates. All participants were categorized into 4 groups according to the age with 5 years interval (26 ~ 30 years, 31 ~ 35 years, 36 ~ 40 years and 41 ~ 45 years, respectively). Age, height and weight were recorded, and height and weight were evaluated by standard methods. Body mass index (BMI) was calculated as weight (kg) divided by height squared (m^2^).

### Image acquisition and analysis

All participants underwent chest CT examinations combined with QCT using 128-slice multidetector CT (Somatom Sensation, Siemens, Erlangen, Germany), and CT scanning dose protocol (LDCT or CDCT) will be self-determined by the participants. Before the participants underwent chest CT examinations, we will verbally inquiry them whether to scan in LDCT scanning protocol or in CDCT scanning protocol. LDCT and CDCT were all scanned by automated tube current modulation, and other CT scanning parameters were as follow: 120 kilovolt (kV), 512 × 512 matrix, 1 mm slice thickness, and 500 mm field of view, and then recorded the effective milliampere seconds (mAs). Radiation dose estimations delivered during CT examinations were retrieved from patient protocols, including volume CT dose index (CTDIvol, mGy) and dose-length-product (DLP, mGy·cm), and then calculated effective dose (ED, mSv). ED was calculated from the DLP multiplying by a dose conversion factor for the chest of 0.014 mSv/(mGy·cm) [13]. $ED=0.014\times \text{D}\text{L}\text{P}$. Delimiting ED by 1.0 mSv, chest CT examinations combined QCT were categorized into 2 groups, LDCT group and CDCT group. LDCT and CDCT images were transferred to a QCT workstation for analysis, and no extra radiation was involved in this analysis.

**Fig. 1:**
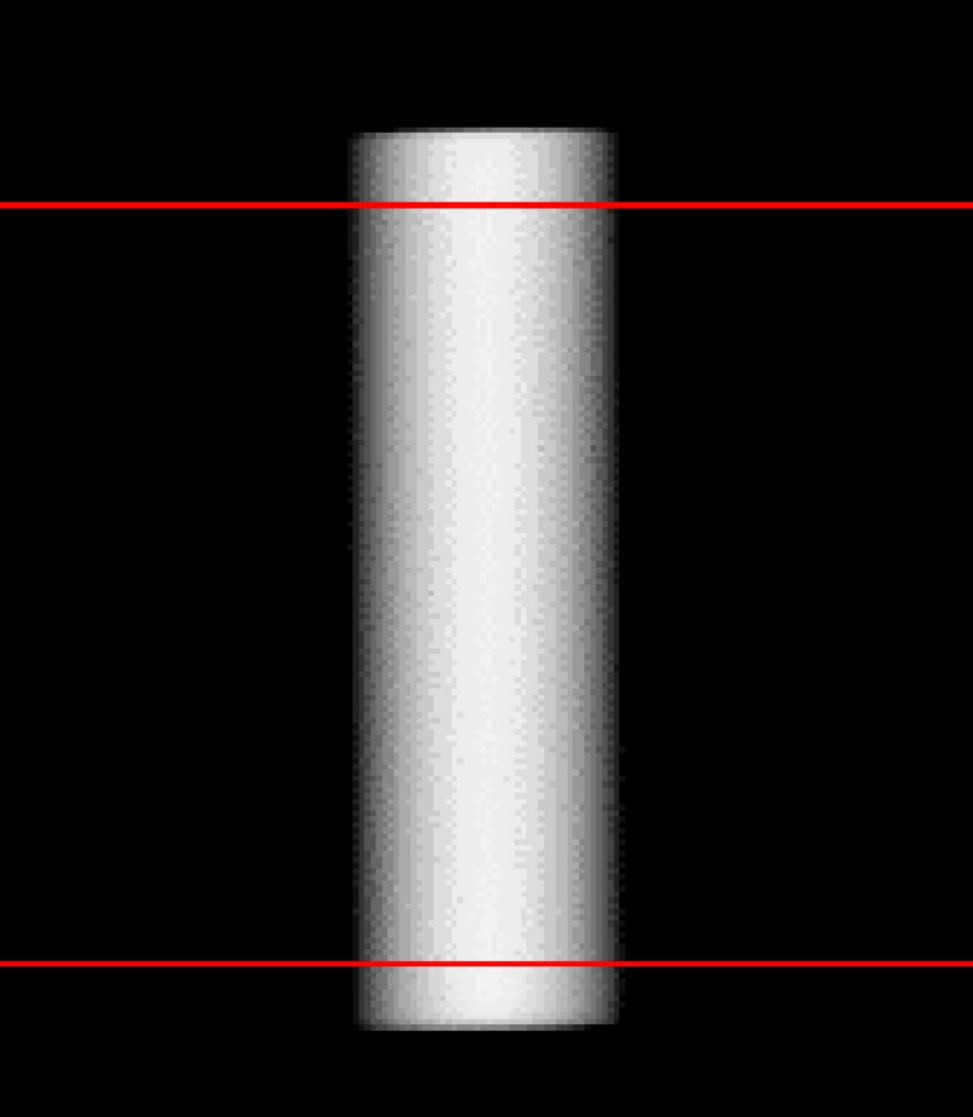
Quality assurance (QA) for asynchronous quantitative computed tomography using the Model 4 asynchronous QA phantom

**Fig. 2:**
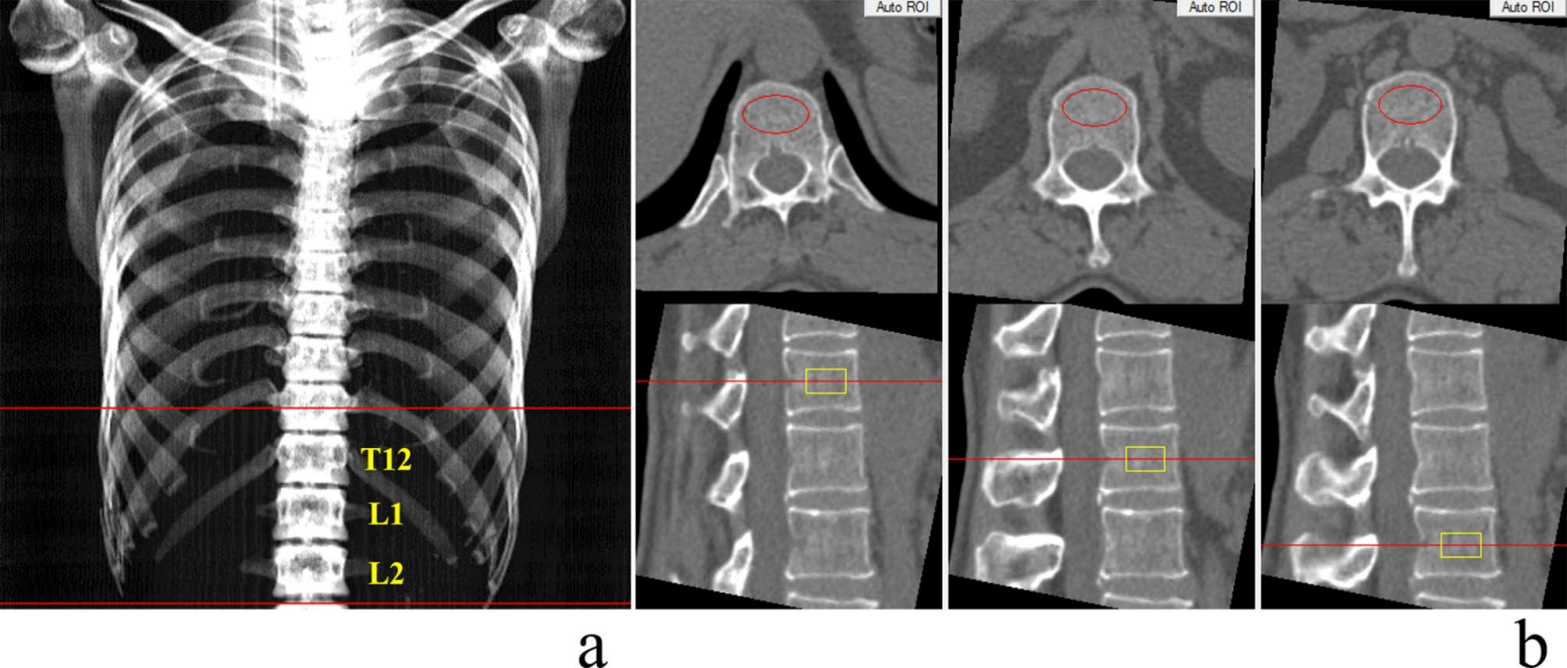
Measurement of vBMD of T12, L1 and L2 with Mindways QCT Pro system. (**a**) Coronal plane of a chest scan. (**b**) Analysis ROIs shown as red ellipse in axial view and yellow rectangle in sagittal view, automatically, avoiding the cortical bone of the vertebrae. ROIs =  regions of interest

Asynchronous vBMD calibration in combination with the QCT Pro analysis software (Mindways Software, Inc.) was used to obtain thoracic and lumbar spine (T12-L2) trabecular vBMD (mg/cm^3^), and a new Model 4 asynchronous calibration phantom (Mindways Software, Inc.) was regularly scanned for quality assurance (QA) calibration (Fig. 1). All analyses were performed by the radiologist who was trained and experienced in using the QCT software for more than 3 years. Because this protocol involved the post-imaging processing of existing plain LDCT or CDCT data, no additional radiation dose was involved. Elliptical regions of interest were put in the midplane of three vertebral bodies (T12-L2) in the trabecular bone automatically, avoiding the cortical bone of the vertebrae automatically (Fig. 2).

### Statistical analysis

Statistical analyses were performed using SPSS version 26.0. Continuous variables were described as mean ± standard deviations or median and quartile spacing. Analysis of variance (ANOVA) was used to compare the difference of vBMD among 4 age groups, and then LSD test for Post Hoc Multiple Comparisons. *Pearson* or *Spearman* test was used to test the correlation between age, BMI, mAs, CTDIvol, DLP and vBMD. Comparison of age, BMI, vBMD, mAs, CTDIvol, DLP and ED between LDCT and CDCT in each age group were used independent samples *t*-test or Mann-Whitney U test respectively. *P* < 0.05 was considered significant.

## Results

### Description characteristics

416 women patients were included, 35.68 ± 4.75 years, from 26 to 45 years (Table 1). There was no difference of vBMD among 4 age groups in LDCT group (*F* = 0.092, *P* = 0.965), and no difference in CDCT group (*F* = 0.044, *P* = 0.988).

**Table 1:** Description characteristics and comparison between LDCT and CDCT

	LDCT group	CDCT group	*t* or *Z*	*P*
26 ~ 30 Years old (*n*)	21	32		
age (years)	28.43 ± 1.21	28.16 ± 1.25	0.787	0.435
BMI (kg/m^2^)	21.41 ± 2.77	20.81 ± 3.22	0.705	0.484
vBMD (mg/cm^3^)	184.26 ± 21.71	181.77 ± 29.96	0.328	0.744
mAs	**16.00 (14.00, 21.00)**	**90.00 (61.25, 116.00)**	**-5.795**	**< 0.001**
CTDIvol (mGy)	**1.12 (0.96, 1.47)**	**6.10 (4.18, 7.85)**	**-5.793**	**< 0.001**
DLP (mGy·cm)	**45.70 (33.00, 54.30)**	**227.05 (165.35, 297.78)**	**-6.111**	**< 0.001**
ED (mSv)	**0.63 ± 0.16**	**3.26 ± 1.14**	**-12.886**	**< 0.001**
31 ~ 35 Years old (*n*)	98	80		
age (years)	33.21 ± 1.36	33.48 ± 1.39	-1.259	0.210
BMI (kg/m^2^)	**21.49 ± 2.65**	**22.99 ± 3.63**	**-3.101**	**0.002**
vBMD (mg/cm^3^)	183.17 ± 27.66	180.27 ± 26.71	0.708	0.480
mAs	**17.00 (13.75, 20.00)**	**94.50 (77.00, 124.25)**	**-11.466**	**< 0.001**
CTDIvol (mGy)	**1.15 (0.95, 1.38)**	**6.41(5.23, 8.41)**	**-11.459**	**< 0.001**
DLP (mGy·cm)	**43.50 (36.10, 53.25)**	**241.45(186.50, 309.48)**	**-11.463**	**< 0.001**
ED (mSv)	**0.64 ± 0.15**	**3.45 ± 1.31**	**-19.017**	**< 0.001**
36 ~ 40 Years old (*n*)	56	53		
age (years)	37.86 ± 1.52	38.15 ± 1.41	-1.046	0.298
BMI (kg/m^2^)	22.31 ± 2.66	23.29 ± 3.64	-1.598	0.113
vBMD (mg/cm^3^)	181.15 ± 26.78	181.13 ± 33.13	0.004	0.997
mAs	**20.00 (17.00, 22.00)**	**88.00 (59.50, 117.00)**	**-9.013**	**< 0.001**
CTDIvol (mGy)	**1.31 (1.20, 1.52)**	**5.96 (4.06, 7.93)**	**-8.998**	**< 0.001**
DLP (mGy·cm)	**50.05 (44.33, 56.80)**	**225.40 (147.75, 291.10)**	**-8.997**	**< 0.001**
ED (mSv)	**0.69 ± 0.15**	**3.21 ± 1.47**	**-12.452**	**< 0.001**
41 ~ 45 Years old (*n*)	41	35		
age (years)	**42.54 ± 1.45**	**43.57 ± 1.17**	**-3.383**	**0.001**
BMI (kg/m^2^)	23.13 ± 2.38	22.62 ± 2.74	0.857	0.394
vBMD (mg/cm^3^)	182.94 ± 31.12	179.45 ± 28.84	0.504	0.616
mAs	**20.00 (15.00, 22.00)**	**96.00 (88.00, 120.00)**	**-7.491**	**< 0.001**
CTDIvol (mGy)	**1.29 (1.07, 1.52)**	**6.49 (5.96, 7.59)**	**-7.480**	**< 0.001**
DLP (mGy·cm)	**48.80 (41.00, 55.55)**	**257.00(222.20, 306.30)**	**-7.478**	**< 0.001**
ED (mSv)	**0.69 ± 0.15**	**3.58 ± 0.97**	**-17.361**	**< 0.001**

LDCT = low-dose computed tomography. CDCT = conventional-dose computed tomography. BMI = body mass index. vBMD = volumetric bone mineral density. mAs = effective milliampere seconds. CTDIvol = volume CT dose index. DLP = dose-length-product

### Correlation between QCT indexes and clinical data

**Fig. 3:**
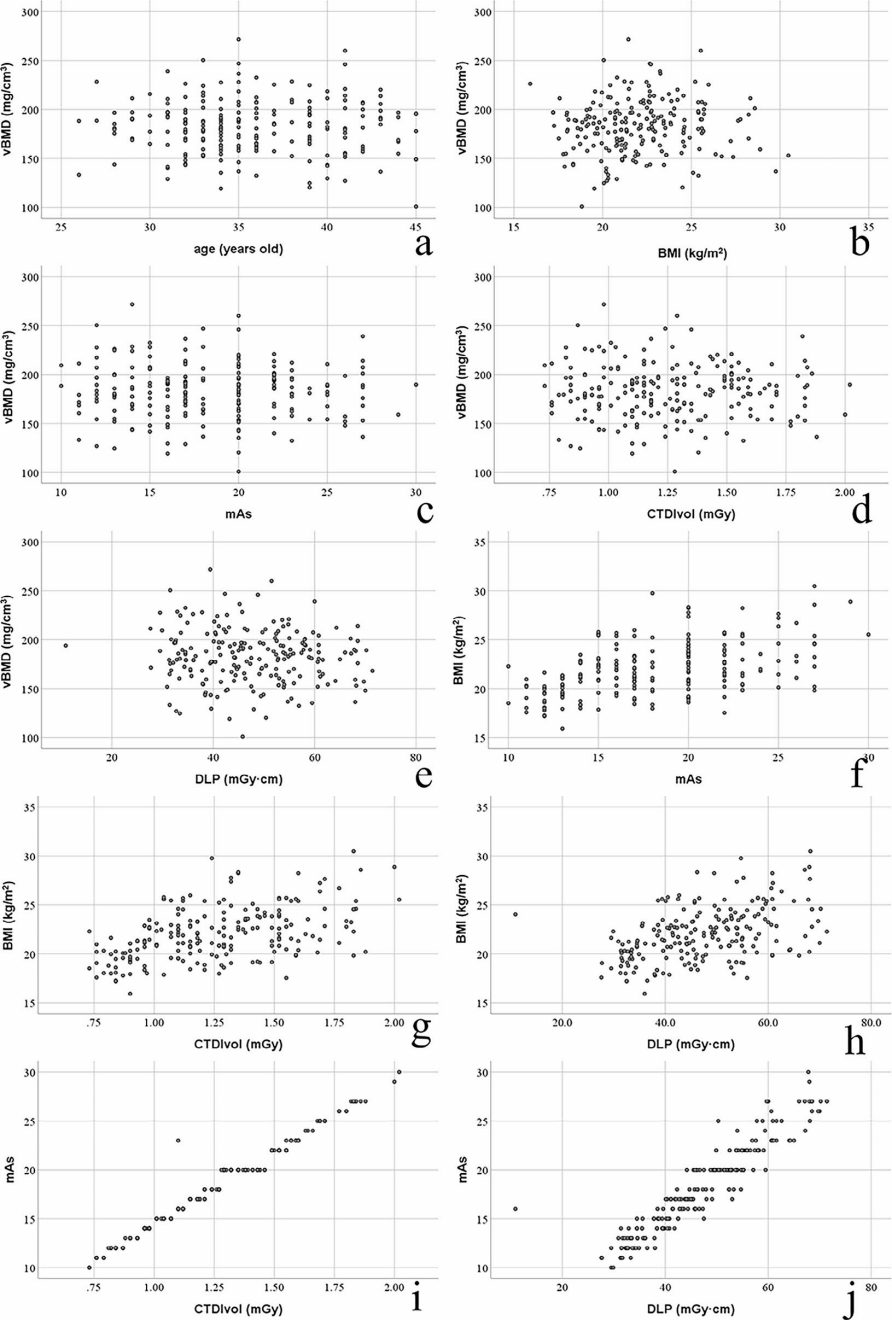
Scatter plots of correlation among parameters in LDCT group. (**a**) Correlation between years and vBMD (*r* = -0.032, *P* = 0.637). (**b**) Correlation between BMI and vBMD (*r* = 0.061, *P* = 0.375). (**c**) Correlation between mAs and vBMD (*r*_*s*_ = -0.013, *P* = 0.850). (**d**) Correlation between CTDIvol and vBMD (*r*_*s*_ = 0.001, *P* = 0.992). (**e**) Correlation between DLP and vBMD (*r*_*s*_ = -0.061, *P* = 0.371). (**f**) Correlation between mAs and BMI (*r*_*s*_ = 0.472, *P* < 0.001). (**g**) Correlation between CTDIvol and BMI (*r*_*s*_ = 0.475, *P* < 0.001). (**h**) Correlation between DLP and BMI (*r*_*s*_ = 0.457, *P* < 0.001). (**i**) Correlation between CTDIvol and mAs (*r*_*s*_ = 0.987, *P* < 0.001). (**j**) Correlation between DLP and mAs (*r*_*s*_ = 0.945, *P* < 0.001)

In LDCT group, vBMD was not correlated to age (*r* = -0.032, *P* = 0.637, Fig. 3a), not correlated to BMI (*r* = 0.061, *P* = 0.375, Fig. 3b), and not correlated to mAs, CTDIvol and DLP (*r*_*s*_ = -0.013, *P* = 0.850; *r*_*s*_ = 0.001, *P* = 0.992 and *r*_*s*_ = -0.061, *P* = 0.371; Fig. 3c, d and e). In LDCT group, BMI was correlated to mAs, CTDIvol and DLP (*r*_*s*_ = 0.472, *P* < 0.001; *r*_*s*_ = 0.475, *P* < 0.001 and *r*_*s*_ = 0.457, *P* < 0.001; Fig. 3f, g and h). In LDCT group, mAs was correlated to CTDIvol and DLP (*r*_*s*_ = 0.987, *P* < 0.001 and *r*_*s*_ = 0.945, *P* < 0.001; Fig. 3i and j).

**Fig. 4:**
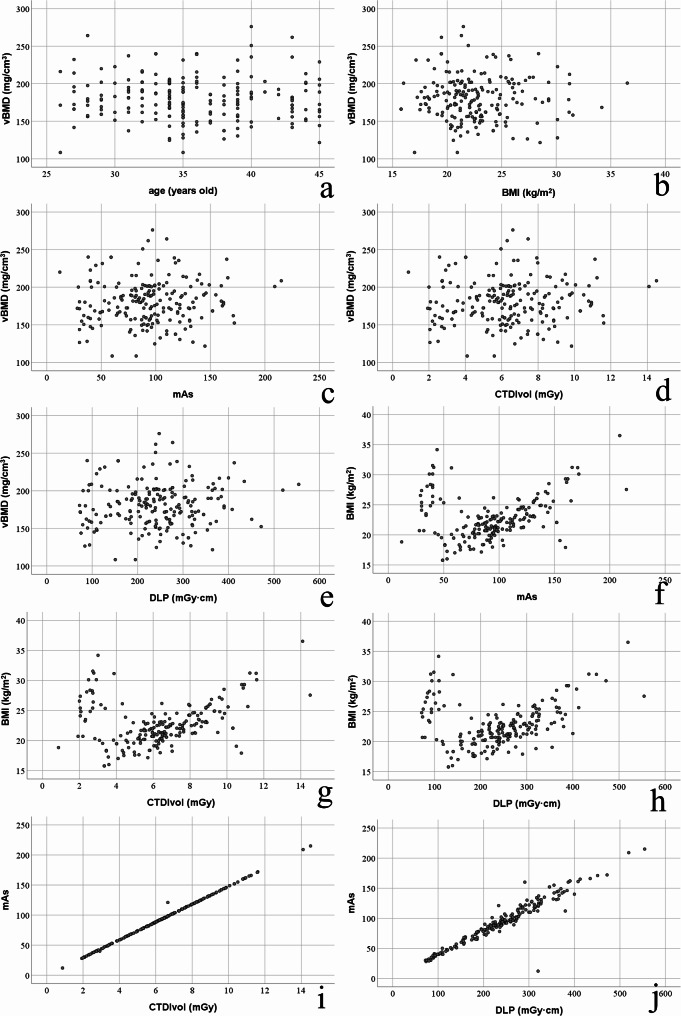
Scatter plots of correlation among parameters in CDCT group. (**a**) Correlation between years and vBMD (*r* = -0.035, *P* = 0.623). (**b**) Correlation between BMI and vBMD (*r* = -0.010, *P* = 0.883). (**c**) Correlation between mAs and vBMD (*r*_*s*_ = 0.068, *P* = 0.341). (**d**) Correlation between CTDIvol and vBMD (*r*_*s*_ = 0.064, *P* = 0.365). (**e**) Correlation between DLP and vBMD (*r*_*s*_ = 0.076, *P* = 0.282). (**f**) Correlation between mAs and BMI (*r*_*s*_ = 0.298, *P* < 0.001). (**g**) Correlation between CTDIvol and BMI (*r*_*s*_ = 0.296, *P* < 0.001). (**h**) Correlation between DLP and BMI (*r*_*s*_ = 0.287, *P* < 0.001). (**i**) Correlation between CTDIvol and mAs (*r*_*s*_ = 0.999, *P* < 0.001). (**j**) Correlation between DLP and mAs (*r*_*s*_ = 0.957, *P* < 0.001)

In CDCT group, vBMD was not correlated to age (*r* = -0.035, *P* = 0.623, Fig. 4a), not correlated to BMI (*r* = -0.010, *P* = 0.883, Fig. 4b), and not correlated to mAs, CTDIvol and DLP (*r*_*s*_ = 0.068, *P* = 0.341; *r*_*s*_ = 0.064, *P* = 0.365 and *r*_*s*_ = 0.076, *P* = 0.282; Fig. 4c, d and e). In CDCT group, BMI was correlated to mAs, CTDIvol and DLP (*r*_*s*_ = 0.298, *P* < 0.001; *r*_*s*_ = 0.296, *P* < 0.001 and *r*_*s*_ = 0.287, *P* < 0.001; Fig. 4f, g and h). In CDCT group, mAs was correlated to CTDIvol and DLP (*r*_*s*_ = 0.999, *P* < 0.001 and *r*_*s*_ = 0.957, *P* < 0.001; Fig. 4i and j).

### Comparison between LDCT and CDCT

**Fig. 5:**
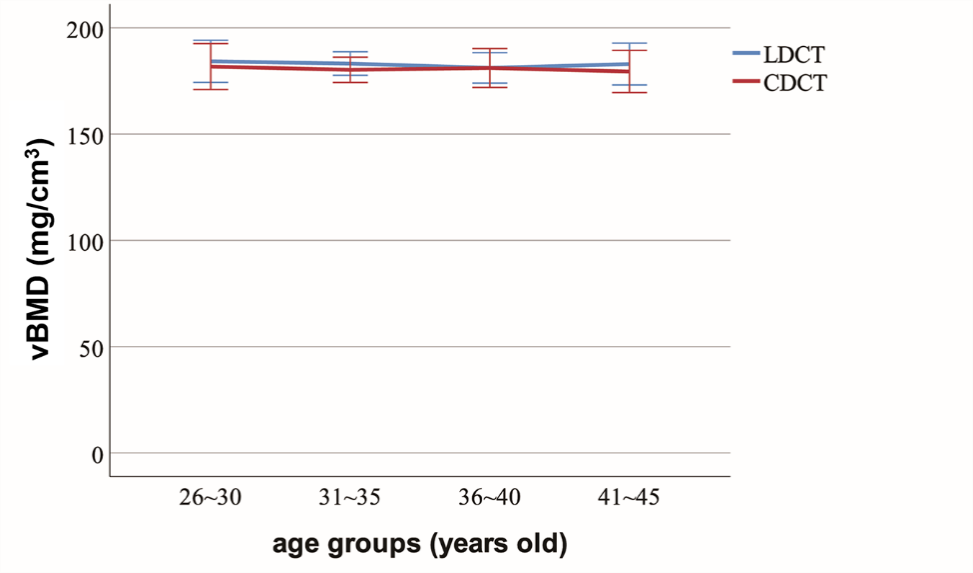
Comparison of vBMD between LDCT and CDCT in different age groups

**Fig. 6:**
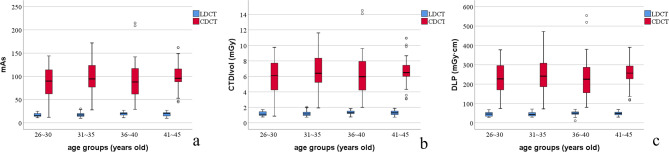
Comparison of mAs, CTDIvol and DLP between LDCT and CDCT in different age groups

Between LDCT and CDCT groups, there was no difference of vBMD (*P* ≥ 0.480, Table 1; Fig. 5), and there were differences of mAs, CTDIvol and DLP (*P* < 0.001, Table 1; Fig. 6).

## Discussion

The traditional mode for vBMD assessment of QCT is to calibrate the Hounsfield Unit (HU) of the lumbar vertebra using a set of reference standards of known vBMD [14]. The vBMD may be assessed by using an internal reference standards, such as the fat and/or muscle tissue [15], or by using an external reference standard, such as a calibrate phantom placed closely dorsal side of each participant during the QCT examination [16]. Asynchronous QCT, a newly developed method, does not require a calibrate phantom to be placed beneath the patient during the QCT scan, while asynchronous QCT need to perform regularly quality assurance (QA) scan, which is an external reference standard QCT system to estimate a ‘‘field-uniformity correction’’ (FUC) for the CT value deficit [17, 18], and we performed QA test daily before the patients underwent QCT examinations.

Previous study showed that vBMD was decreased with age [1].The loss of BMD in old women includes two stages, a slow constant age-related loss and a quick oestrogen-dependent process. Ageing, hormonal imbalance, environmental factors and genetic predispositions are all responsible for BMD loss [19]. Not consistent with above results, in present study, there were no difference of vBMD among 4 age groups and vBMD not correlated to age in both LDCT group and CDCT group. This may be that the enrolled population of this study was 25 ~ 45 years old female and previous studies have shown that the peak age of vBMD in women was from 30 to 39 years old and all subjects were premenopausal, which the quick process of hormone-oestrogen-dependent vBMD loss has not yet begun [1].

Many mechanisms exist for the effects of fat on bone, including the effect of soft tissue mass on bone load, fat mass and the secretion of bone active hormones (including insulin, amylin and preprotein), and the secretion of bone active hormones (such as estrogen and leptin) from adipocytes. Previous studies have suggested that adipose tissue may affect the vBMD either through the production of hormones and adipokines by adipocytes, or by affecting the secretion of active bone sex hormones by the pancreas [20]. Adipose tissue is a complex, essential, and highly active metabolic and endocrine organ. In addition to adipocytes, adipose tissue contains the connective tissue matrix, neural tissue, stromovascular cells, and immune cells. Adipose tissue is metabolically heterogeneous, with subcutaneous adipose and visceral adipose tissue, while BMI cannot adequately distinguish [21]. In present study, vBMD was not correlated to BMI in both LDCT group and CDCT group.

In a study by Wu Y et al. [10], radiation dose (CTDIvol and DLP) was positively correlated with tube current, which was consistent with the results of the previous study. Our study also demonstrated that mAs was correlated to CTDIvol and DLP in both LDCT group and CDCT group. Previous study showed that radiation dose had a strong positive correlation with both BMI [22], and our study also showed that BMI was correlated to CTDIvol and DLP in both LDCT group and CDCT group. These results suggest that higher BMI, about higher mAs and higher radiation dose.

QCT involves converting HU to values of vBMD through using a calibration reference phantom or not [5, 23]. Many factors during the CT scan can affect the HU values, such as beam hardening in the x-ray energy spectrum. Different scanners or the same scanner at different times could measure varying HU values, although modern scanner calibration techniques greatly reduce this effect [23]. In a study by Giambini H et al., a rabbit femur and a standard calibration phantom were imaged by QCT using different protocols, and vBMD were affected by voltage and kernel but not by current [24]. In present study, the difference of CT scanning dose protocol between LDCT and CDCT group) was only the difference in mAs, and we used the same scanners, kV and other parameters. Consistent with the previous study, our study demonstrated that vBMD was not correlated to mAs and there was no difference of vBMD between LDCT group and CDCT group.

### Limitations

Our study has several limitations. First, only 26 ~ 45 years old women were included in the research and the sample size of 26 ~ 30 years old women was smaller. The vBMD distribution of male and female population is different at different ages and the sample size of male participants in this study is small, we only analyzed the vBMD of the female sample in patient population. Thus, additional, large sample size and multicenter studies are required to independently validate these results. Second, CT scanning dose protocol (LDCT or CDCT) was self-determined by the participant, and automated tube current modulation was selected not fixed mAs. We used the automated tube current modulation rather than the fixed mA. Along with vBMD measurement, the participants also need to be tested for lesions in their lungs, and Siemens CT equipment now almost all use ACTM, which is able to obtain stable image quality in various body types and body parts. The fixed mA will increase the image noise due to insufficient exposure dose in larger body sizes participants, which will affect the image diagnosis. However, in the smaller body sizes participants, although the image quality is not affected, the exposure dose is increased. Final, the value of our reproducibility data was limited because vBMD was only measured by one radiologist.

## Conclusion

We demonstrated that vBMD was not correlated to mAs and there was no difference of vBMD between LDCT group and CDCT group combined with asynchronous QCT. While screening for diseases such as lung cancer and mediastinal lesions, LDCT combined with asynchronous QCT can be also used to assess vBMD simultaneously with no extra imaging equipment, patient visit time, radiation dose and no additional economic cost.

### Electronic supplementary material

Below is the link to the electronic supplementary material.

Supplementary Material 1

## Data Availability

All data generated and analyzed during this study are included in this published article and its supplementary information file.

## Abbreviations

vBMD: Volumetric bone mineral density
mg/cm^3^: Milligrams per cubic centimeter
LDCT: Low-dose CT
CTDIvol: Volume CT dose index
ED: Effective dose
ANOVA: Analysis of variance
Asynchronous QCT: Asynchronous calibration QCT
CDCT: Conventional-dose CT
ESP: European spine phantom
DLP: Dose-length-product
QA: Quality assurance
HU: Hounsfield Unit
FUC: Field-uniformity correction
